# Development of tissue inflammation accompanied by NLRP3 inflammasome activation in rabbits infected with *Treponema pallidum strain Nichols*

**DOI:** 10.1186/s12879-018-2993-0

**Published:** 2018-03-01

**Authors:** Li-Rong Lin, Yao Xiao, Wei Liu, Yu-Yan Chen, Xiao-Zhen Zhu, Zheng-Xiang Gao, Kun Gao, Man-Li Tong, Hui-Lin Zhang, Shu-Lian Li, Hui-Ling Lin, Wen-Dong Li, Xian-Ming Liang, Yong Lin, Li-Li Liu, Tian-Ci Yang

**Affiliations:** 1 Zhongshan Hospital, Medical College of Xiamen University, Xiamen, Fujian Province China; 2 Institute of Infectious Disease, Medical College of Xiamen University, Xiamen, Fujian Province China; 3 Xiamen Hospital of Traditional Chinese Medicine, Xiamen, Fujian Province China; 4 Xiamen Fifth Hospital, Xiamen, Fujian Province China; 5 Xiamen Huli District Maternity and Child Care Hospital, Xiamen, Fujian Province China

**Keywords:** *Treponema pallidum*, Inflammation, NLRP3, Il-1β, Rabbit

## Abstract

**Background:**

The inflammasome responses in *Treponema pallidum* infection have been poorly understood to date. This study aimed to investigate the expression of the nucleotide-binding leucine-rich receptor protein 3 (NLRP3) inflammasome in the development of tissue inflammation in rabbits infected with *T. pallidum*.

**Methods:**

Forty-five rabbits were randomly assigned to a blank group or an infection group, and the latter was divided into no benzathine penicillin G (BPG) and BPG treatment subgroups. Rabbits in the infection group were injected intradermally with 0.1 mL of a 10^7^/mL *T. pallidum* suspension at 10 marked sites along the back, and the blank group was treated with normal saline. The BPG treatment subgroup received 200,000 U of BPG administered intramuscularly twice, at 14 d and 21 d post-infection. The development of lesions was observed, and biopsies of the injection site and various organs, including the kidney, liver, spleen, lung, and testis, were obtained for *NLRP3, caspase-1, and interleukin-1β (IL-1β)* mRNA analysis during infection. Blood was also collected for the determination of IL-1β concentration.

**Results:**

Rabbits infected with *T. pallidum* (both the BPG treatment and no BPG treatment subgroups), exhibited NLRP3 inflammasome activation and IL-1β secretion in cutaneous lesions, showing a trend in elevation to decline; *NLRP3* mRNA expression reached a peak at 18 d in the BPG treatment subgroup and 21 d in the no BPG treatment subgroup and returned to “normal” levels [vs. the blank group (*P* > 0.05)] at 42 d post-infection. The trend was similar to the change in cutaneous lesions in the infected rabbits, which reached a peak at 16 d in the BPG treatment subgroup and 18 d in the no BPG treatment subgroup. *NLRP3, caspase-1,* and *IL-1β* mRNA expression levels were slightly different in different organs. NLRP3 inflammasome activation was also observed in the kidney, liver, lung, spleen and testis. *IL-1β* expression was observed in the kidney, liver, lung and spleen; however, there was no detectable level of *IL-1β* in the testes of the infected rabbits.

**Conclusions:**

This study established a clear link between NLRP3 inflammasome activation and the development of tissue inflammation in rabbits infected with *T. pallidum*. BPG therapy imperceptibly adjusted syphilitic inflammation.

## Background

Syphilis is a sexually transmitted disease caused by the bacterial spirochete *Treponema pallidum* [1]. The inflammatory processes induced by *T. pallidum* within infected tissues result in the development of lesions, and lesion resolution has been reported previously [2]. The innate immune system, the first line of host defense of microbial infection, is recognized as the major contributor to the acute inflammation induced by tissue damage or microbial infection [3]. The innate immune system has an imperative function in controlling the initial pathogen invasion and activates various members of the nucleotide-binding leucine-rich receptor (NLR) family in the cytoplasm, resulting in the assembly of an NLR-containing multiprotein complex that recruits and activates caspase-1, leading to interleukin-1β (IL-1β) production [4].

NLRP3 is the best-characterized member of the NLR family involved in the innate immune system; this system is activated by exogenous and endogenous stimulatory factors, such as bacteria, viruses, fungi, and components of dying cells [5, 6], and NLRP3 serves as a platform for the activation of caspase-1 and the maturation of the pro-inflammatory cytokine IL-1β to engage in the innate immune response [7]. The role of the NLRP3 inflammasome in pathogenic infections, such as those caused by *Pneumococcus* [8], *Helicobacter pylori* [9], *Neospora caninum* [10], and *Mycobacterium tuberculosis* [11] has been demonstrated. However, the involvement of NLRP3 in the inflammatory processes of *T. pallidum* infection is poorly understood.

In this study, we investigate the expression of the NLRP3 inflammasome during the development of tissue inflammation associated with syphilis, the activation of the inflammasome and release of IL-1β were estimated during *T. pallidum* infection in a rabbit model.

## Methods

### Animal experiments

The *T. pallidum* Nichols strain was kindly provided by Lorenzo Giacani, Ph.D. (University of Washington, Seattle) and was propagated via intra-testicular serial passage in New Zealand white rabbits to maintain virulence in our laboratory as previously described [12]. Forty-five male New Zealand white rabbits (purchased from the Xiamen University Laboratory Animal Center, weighing approximately three kilograms each) with negative results in both the reactive rapid plasma reagin and *T. pallidum* particle agglutination tests, were randomly assigned to two groups, a blank group (*n* = 15) and an infection group (*n* = 30). The latter was divided into the no benzathine penicillin G (BPG) treatment subgroup (*n* = 15) and the BPG treatment subgroup (*n* = 15). The animals were housed individually at 16 to 18 °C and were fed with antibiotic-free food and water. Rabbits in the infection group were injected intradermally with 0.1 mL of a 10^7^ treponeme/mL suspension at 10 marked sites along the back, while rabbits in the blank group were injected with normal saline. The backs of the rabbits were meticulously kept free of fur by daily clipping throughout the experiment. Rabbits in the BPG treatment subgroup received 200,000 U of BPG administered intramuscularly twice, at 14 d and 21 d post-infection.

One representative site of each animal was selected separately and biopsied (4-mm punch biopsies obtained under local lidocaine anesthesia) for RNA extraction at 1, 4, 7, 10, 14, 18, 21, 28, 35 and 42 d post-infection. One representative site on each animal was dedicated exclusively for the observation of lesion appearance and development up to 42 d post-infection; the diameter of the lesion was measured using a vernier caliper. Three animals were randomly selected for euthanasia in the two groups at 7, 14, 21, 28, and 42 d post-infection, and the kidney, liver, spleen, lung, and testis organs were then harvested for experimental analysis. Blood was collected at 1, 4, 7, 10, 14, 18, 21, 28, 35 and 42 d post-infection, and serum was isolated and frozen at − 80 °C until analysis of the IL-1β concentration. All protocols involving animals were approved in advance by the animal experimental ethics committee of the Medical College of Xiamen University.

### *NLRP3/caspase-1/ IL-1β* mRNA expression analysis

To assess the expression of mRNA, total RNA from lesions/tissues was isolated using the RNeasy Kit (Qiagen Inc., Valencia, CA) and was reverse transcribed using a high-capacity cDNA reverse transcription kit (Takara Inc., Dalian, China). The generated cDNA was amplified using quantitative PCR assays and the SYBR Advantage PCR Premix (Takara Inc., Dalian, China) with the 7500 Real Time PCR System (Applied Biosystems, Carlsbad, USA). The following primer pairs were used: *NLRP3*, (5’-CCACTTCCCCAGAATCGAGA*-*3′ and 5’-TGGACGTGAGACAGGAGTTC*-*3′); *Caspase-1,* (5’-CAAGTCTCAAGCTTTGCCCG*-*3′ and 5’-TAATGAGGGCAAGACGGGTG*-*3′); *IL-1β*, (5’-GGATGACGGCCTGAGAACTT*-*3′ and 5’-TACGTGCCAGACAACACCAA*-*3′); and *GAPDH,* (5’-GCTTCTTCTCGTGCAGTGCA*-*3′ and 5’-ATGACCAGCTTCCCGTTCTC *-*3′). After amplification, Ct values were normalized to *GAPDH* as an internal control, and the relative copy number was determined using the standard 2^-△△Ct^ method [13]. A commercial enzyme-linked immunosorbent assay kit (Cloud-Clone Inc., USA) was used to measure the IL-1β levels in the rabbit serum samples according to the manufacturer’s instructions.

### Statistical analysis

The data were expressed as the mean ± SD. Statistical analyses were performed using the SPSS 13.0 software (SPSS Inc., Chicago, USA). Student’s t-test was applied to compare the means between two groups. In cases with more than two groups, a one-way analysis of variance was employed to examine the differences between the groups, and Dunnett’s post-comparison test was used to conduct multiple comparisons. A 2-tailed *P* value of less than 0.05 was accepted as being statistically significant.

## Results

### Development of cutaneous lesions in rabbits infected with *T. pallidum*

**Fig. 1:**
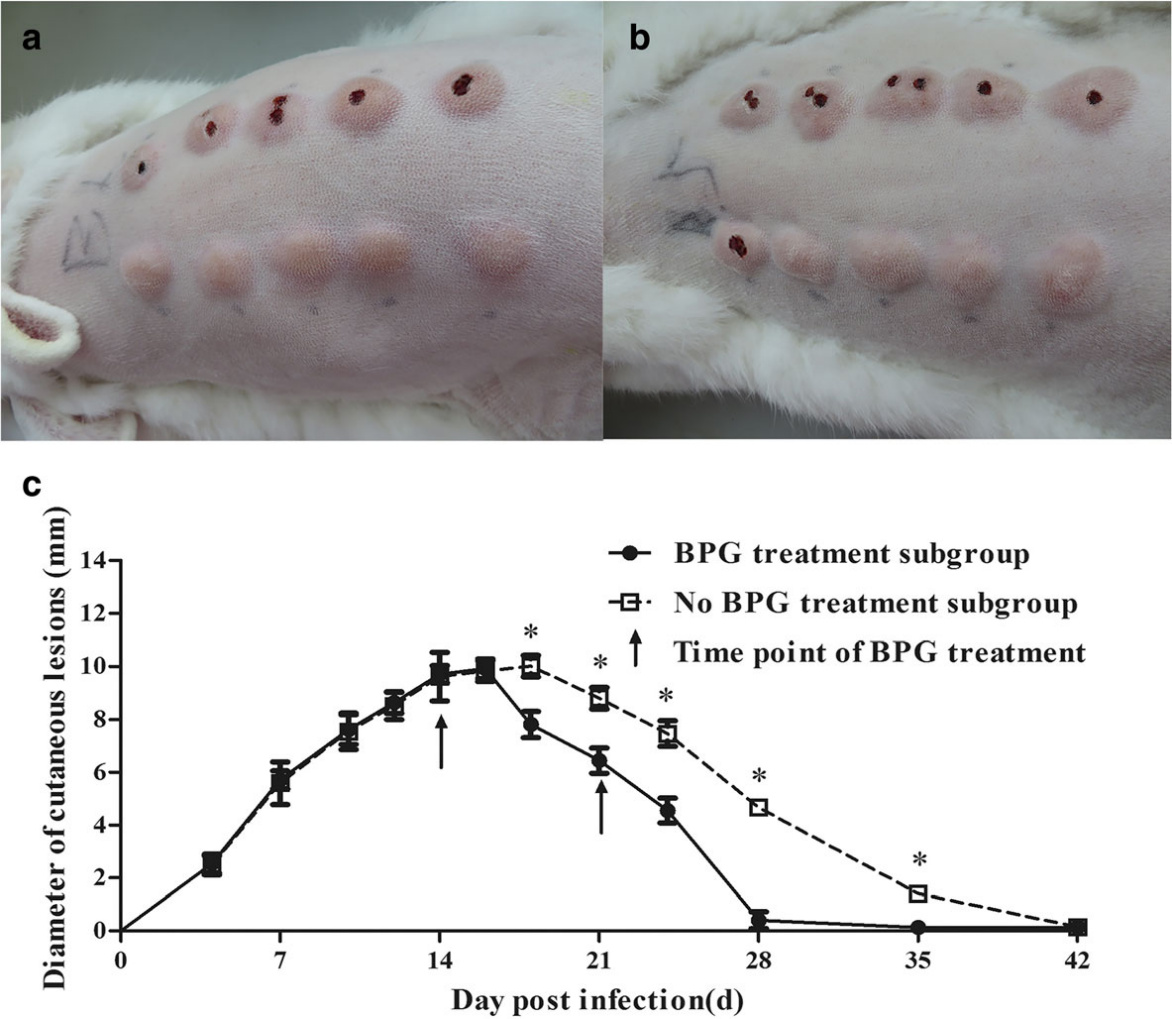
Dynamics of the cutaneous lesion size in rabbits infected with *T. pallidum*. **a** Lesions of one representative rabbit in the BPG treatment subgroup at 16 d. **b** Lesions of one representative rabbit in the no BPG treatment subgroup at 18 d. **c** Dynamics of the cutaneous lesion size in rabbits infected with *T. pallidum*. The arrow shows the time point of BPG treatment. The results are expressed as the mean ± SD. Student’s *t*-test was applied to compare the means of the diameters between the BPG treatment and no BPG treatment subgroups. ^*^P < 0.05

In the infected rabbits, cutaneous lesions began to develop at 4 d post-infection and then reached a peak at 16 d in the BPG treatment subgroup and at 18 d in the no BPG treatment subgroup (Fig. 1a, b). In the BPG treatment subgroup, the lesions gradually began to shrink at 16 d (2 d after the first BPG treatment) and subsequently disappeared at 28 d post-infection. The cutaneous lesions disappeared at an earlier time point in the BPG treatment subgroup than that in the no BPG treatment subgroup (28 d vs. 42 d). The lesions were barely detectable at 35 d and disappeared at 42 d in the no BPG treatment subgroup. The cutaneous lesions in the no BPG treatment subgroup were significantly larger than those in the BPG treatment subgroup at 18, 21, 24, 28 and 35 d post-infection (Fig. 1c) (*P* < 0.05). No lesions developed in the blank group (data not shown).

### NLRP3 inflammasome activation in the cutaneous lesions of infected rabbits

**Fig. 2:**
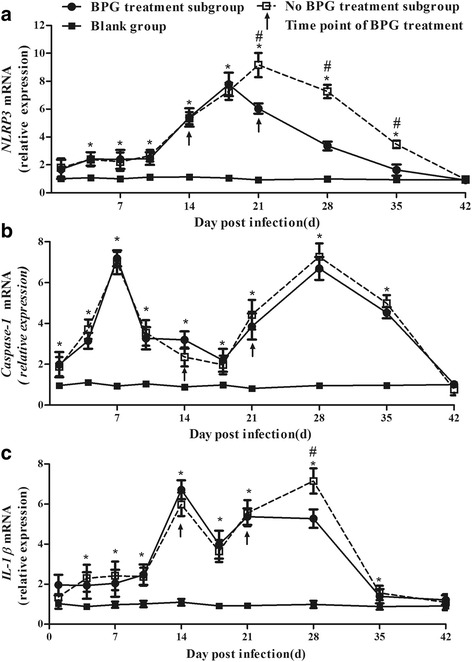
Dynamics of *NLRP3, caspase-1, and IL-1β* mRNA expression in cutaneous lesions in rabbits infected with *T. pallidum.* **a** Dynamics of *NLRP3* mRNA in cutaneous lesions in rabbits infected with *T. pallidum*. **b** Dynamics of *caspase-1* mRNA in cutaneous lesions in rabbits infected with *T. pallidum*. **c** Dynamics of *IL-1β* mRNA in cutaneous lesions in rabbits infected with *T. pallidum*. The arrow shows the time point of BPG treatment. Values represent the mean ± SD of triplicate experiments. A one-way analysis of variance was employed to examine the differences in the three groups, and Dunnett’s post-comparison test was used to conduct multiple comparisons. * *P* < 0.05, the BPG treatment subgroup or no BPG treatment subgroup vs. the blank group. # *P* < 0.05, the BPG treatment subgroup vs. the no BPG treatment subgroup

In the infected rabbit group, the *NLRP3* mRNA levels showed a trend in elevation to decline and reached a peak at 18 d post-infection in the BPG treatment subgroup and at 21 d in the no BPG treatment subgroup. In the BPG treatment subgroup, *NLRP3* mRNA expression was suppressed at 18 d post-infection (4 d after the first BPG treatment) and returned to “normal” levels [(i.e., not significantly different from the blank group (*P* > 0.05)] at 42 d post-infection. Notably, the level of *NLRP3* mRNA exhibited a reduction at an earlier time point in the BPG treatment subgroup (18 d) than that in the no BPG treatment subgroup (21 d). The expression of *NLRP3* mRNA in the no BPG treatment subgroup was significantly higher than that in the BPG treatment subgroup at 21, 28, and 35 d post-infection (*P* < 0.05) (Fig. 2a). However, the expression of *caspase-1* and *IL-1β* mRNA showed a “saddle pattern” of change over time post-infection; *caspase-1* expression reached an initial peak at 7 d and a second peak at 28 d, while *IL-1β* mRNA reached a first peak at 14 d and a second peak at 28 d in both the BPG treatment and no BPG treatment subgroups (Fig. 2b, c). Notably, despite different trends in the expression of *NLRP3*, *caspase-1*, and *IL-1β* mRNAs in cutaneous lesions during infection, at 42 d post-infection, the expression of all three mRNAs returned to “normal” levels [i.e., were not significantly different from the blank group levels (*P* > 0.05)] in both the BPG treatment and no BPG treatment subgroups. The expression of *NLRP3*, *caspase-1*, and *IL-1β* mRNAs was maintained at a low level and showed no fluctuations in the blank group during the experimental period (Fig. 2).

### IL-1β secretion in rabbits infected with *T. pallidum*

**Fig. 3:**
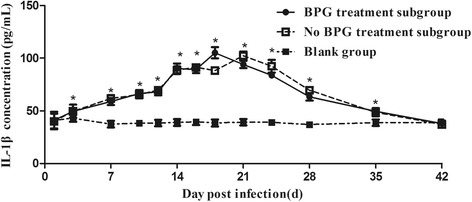
Dynamics of the IL-1β concentration in rabbits infected with *T. pallidum.* Values represent the mean ± SD of triplicate experiments. A one-way analysis of variance was employed to examine the differences between groups, and Dunnett’s post-comparison test was used to conduct multiple comparisons. * *P* < 0.05, the BPG treatment subgroup or no BPG treatment subgroup vs. the blank group

Except at 1 d and 42 d post-infection, the IL-1β concentration in the infection group was significantly higher than that in the blank group, which maintained a low level with no fluctuations. The dynamic tendency of the serum IL-1β concentration in the BPG treatment subgroup was similar to that in the no BPG treatment subgroup, which presented a trend of increasing early and a later decrease (Fig. 3). The serum IL-1β level reached a peak at 18 d in the BPG treatment subgroup and at 21 d in the no BPG treatment subgroup, before returning to normal levels [i.e., not significantly different from the blank group level (*P* > 0.05)] at 42 d post-infection.

### NLRP3 inflammasome expression in the organs of rabbits infected with *T. pallidum*

**Fig. 4:**
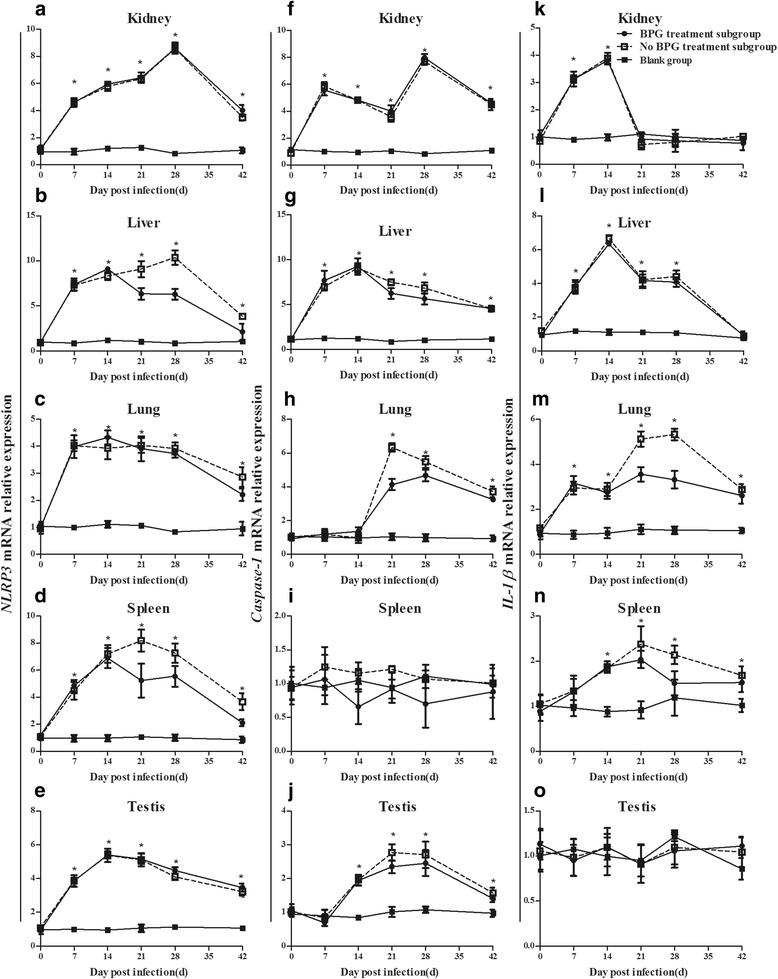
Dynamics of NLRP3, caspase-1, and IL-1β mRNA expression in organs of rabbits infected with *T. pallidum.* **a-e** Dynamics of *NLRP3* mRNA expression in the kidney (**a**), liver (**b**), lung (**c**), spleen (**d**) and testis (**e**). **f-j** Dynamics of *caspase-1* mRNA expression in the kidney (**f**), liver (**g**), lung (**h**), spleen (**i**) and testis (**j**). **k-o** Dynamics of *IL-1β* mRNA expression in the kidney (**k**), liver (**l**), lung (**m**), spleen (**n**) and testis (**o**). Values represent the mean ± SD of triplicate experiments. A one-way analysis of variance was employed to examine the differences in groups, and Dunnett’s post-comparison test was used to conduct multiple comparisons. * *P* < 0.05, the BPG treatment subgroup or no BPG treatment subgroup vs. the blank group

Additionally, the dynamics of *NLRP3*, *caspase-1*, and *IL-1β* mRNAs were monitored in five organs: the kidney, liver, spleen, lung, and testis. The results showed that *NLRP3*, *caspase-1*, and *IL-1β* mRNAs had different expression levels in the different organs of infected rabbits. The *NLRP3* mRNA expression levels in the infection group showed a trend in elevation to decline in all five organs but was still higher than “normal” at the endpoint of the study (vs. the blank group, *P* < 0.05) (Fig. 4a-e). Similar to the trend in *NLRP3*, *caspase-1* mRNA showed an initial increase and then a decreasing trend later in four organs (kidney, liver, lung, and testis). The expression of *caspase-1* mRNA in the kidney showed a “saddle pattern” of change over time post-infection, which was different from that in the other three organs (liver, lung, and testis) and remained higher than that in the blank group at the endpoint of the study (*P* < 0.05). However, *caspase-1* mRNA level in the spleen was not different among the BPG treatment subgroup, the no BPG treatment subgroup, and the blank group (Fig. 4f-j). Regarding the expression level of *IL-1β* mRNA, there was no difference in the testes among the BPG treatment subgroup, the no BPG treatment subgroup, and the blank group. However, *IL-1β* mRNA expression showed an earlier increase and later decrease in the kidney, liver, spleen, and lung and returned to normal at 21 d in the kidney and 42 d in the liver [vs. the blank group (*P* > 0.05)]. *IL-1β* mRNA was still expressed in the lung and spleen (vs. the blank group, *P* < 0.05) at the endpoint of the study (42 d post-infection) (Fig. 4k-o).

## Discussions

*T. pallidum* can provoke an intense innate immune response, which is generally believed to be the cause of tissue damage [14]. In a rabbit model, *T. pallidum* infection presents with the progression of macrophage activation and mononuclear cell infiltration at the sites of the experimental inoculation [15]. Immunohistochemistry and real time-PCR analysis of biopsy specimens obtained from primary and secondary syphilis lesions demonstrate that syphilitic skin lesions are also composed of macrophages and lymphocytes that express mRNAs for *IL-1β, Interferon-γ* and *IL-12* in experimentally infected rabbit tissues [16] and human primary syphilitic lesions [17]. Results from prior studies have confirmed that innate immune cells, such as macrophages, can express pattern recognition receptors and sense microbes by recognizing the pathogen-associated molecular patterns of pathogens [18]; then various members of the NLR family in the cytoplasm are activated, resulting in the assembly of an NLR and the activation of caspase-1, leading to IL-1β production [4]. NLRP3 inflammasome activation/IL-1β release results in hepatocyte pyroptosis, liver inflammation, and fibrosis in mice [19]. In the present study, we found that NLRP3 inflammasome activation and IL-1β secretion were exhibited in *T. pallidum*-infected rabbits at the early phase and showed a trend in elevation to decline. The trend was similar to the changes in the lesions of the infected rabbits, which showed evidence of a link between NLRP3 inflammasome activation and inflammatory injury caused by the *T. pallidum* infection. The activation of the NLRP3 inflammasome is closely related to disease development.

Penicillin has been recommended as the mainstay of treatment for all types of syphilis since this drug was first used for this indication in 1943 [20]. In this study, we also investigated the effect of penicillin treatment on the expression of the NLRP3 inflammasome during the development of tissue inflammation due to syphilis. We found that regardless of whether the infected rabbits received BPG treatment, the expression levels of *NLRP3, caspase-1,* and *IL-1β* in cutaneous lesions all showed an identical trend in elevation to decline, similar to the trend found in the cutaneous lesions, and the expression of *NLRP3*, *caspase-1*, and *IL-1β* mRNAs in lesions eventually returned to “normal” levels in both the BPG treatment and no BPG treatment subgroups, but the time point of reduction was slightly different. The cutaneous lesions disappeared at an earlier time point (at 28 d) in the BPG treatment subgroup than in the no BPG treatment subgroup (at 42 d). In addition, *NLRP3* mRNA expression was suppressed at an earlier time point in the BPG treatment subgroup (18 d) than in the no BPG treatment subgroup (21 d). BPG therapy imperceptibly adjusted syphilitic inflammation.

*T. pallidum* disseminates systemically and induces inflammation in diverse tissues and organs [21]. Innate immune cells, such as macrophages in tissues and organs not only mediate bacterial clearance but also lead to tissue damage and clinical symptoms [22]. In this study, we detected NLRP3 inflammasome activation in five organs, the kidney, liver, lung, spleen and testis, further confirming that *T. pallidum* induced systemic inflammatory during infection. We also found that *IL-1β* was expressed in the kidney, liver, lung and spleen tissue but was not detectable in the testes of the infected rabbits. One possible explanation is that there may be some difference in the number of IL-1β-producing cells (such as macrophages) or in the cellular function cytokine production in response to *T. pallidum* stimulation among different organs, The other possible reason is that the testis represents a distinct immunoprivileged site where invading pathogens can be tolerated without evoking detrimental immune responses [23]. In addition, we found that *NLRP3* was differently expressed in different organs and was also recovered at different times, further confirming the existence of different immune response profiles to *T. pallidum* in different organs. Additionally, only three animals were harvested for experimental analysis; thus, the possibility of individual differences in the immune response of animals may result in the non-regularity. Further study requires more animals to eliminate individual differences.

In this study, we demonstrated that *T. pallidum*-induced inflammasome activation was positively correlated with changes in the skin lesions of rabbits. Further studies are required to understand the mechanisms of NLRP3 inflammasome regulation by IL-1β in *T. pallidum* infection*.* Also, *T. pallidum* multiplicity may correlate with the different disease outcome [1, 24], the immune response to different *T. pallidum* strains would deserve our future study. In addition, we only monitored the changes at 42 d post-infection. Therefore, further studies are required to determine changes in the NLRP3 inflammasome in rabbits with relapse in the no BPG treatment subgroup.

## Conclusions

In the present study, we established a clear link between NLRP3 inflammasome activation and the development of tissue inflammation in rabbits infected with *T. pallidum*; NLRP3 inflammasome activation was similar to the process of self-limited disease. We also found that BPG therapy imperceptibly altered the syphilitic inflammation, but the underlying mechanism remains unclear.

## Abbreviations

BPG: Benzathine penicillin G; IL-1β: Interleukin-1β
NLRP3: Nucleotide-binding leucine-rich receptor protein 3

## References

[1] Tong ML, Zhao Q, Liu LL, Zhu XZ, Gao K, Zhang HL, Lin LR, Niu JJ, Ji ZL, Yang TC (2017). Whole genome sequence of the Treponema pallidum subsp. pallidum strain Amoy: An Asian isolate highly similar to SS14. Plos One.

[2] Salazar JC, Hazlett KR, Radolf JD (2002). The immune response to infection with Treponema pallidum, the stealth pathogen. Microbes & Infection.

[3] Weissleder R, Nahrendorf M, Pittet MJ (2014). Imaging macrophages with nanoparticles. Nat Mater.

[4] Leemans JC, Cassel SL, Sutterwala FS (2011). Sensing damage by the NLRP3 inflammasome. Immunol Rev.

[5] Gross O, Thomas CJ, Guarda G, Tschopp J (2011). The inflammasome: an integrated view. Immunol Rev.

[6] Wang Y, Wang GZ, Rabinovitch PS, Tabas I (2014). Macrophage mitochondrial oxidative stress promotes atherosclerosis and nuclear factor-kappaB-mediated inflammation in macrophages. Circ Res.

[7] Zhang F, Wang L, Wang JJ, Luo PF, Wang XT, Xia ZF (2016). The caspase-1 inhibitor AC-YVAD-CMK attenuates acute gastric injury in mice: involvement of silencing NLRP3 inflammasome activities. Sci Rep.

[8] Geldhoff M, Mook-Kanamori BB, Brouwer MC, Troost D, Leemans JC, Flavell RA, Van der Ende A, Van der Poll T, Van de Beek D (2013). Inflammasome activation mediates inflammation and outcome in humans and mice with pneumococcal meningitis. BMC Infect Dis.

[9] Perez-Figueroa E, Torres J, Sanchez-Zauco N, Contreras-Ramos A, Alvarez-Arellano L, Maldonado-Bernal C (2016). Activation of NLRP3 inflammasome in human neutrophils by helicobacter pylori infection. Innate Immun.

[10] Wang XC, Gong PT, Zhang X, Wang JL, Tai LX, Wang X, Wei ZK, Yang YJ, Yang ZT, Li JH, et al. NLRP3 inflammasome activation in murine macrophages caused by Neospora caninum infection. Parasit Vectors. 2017;10(266):1-13. https://rd.springer.com/content/pdf/10.1186%2Fs13071-017-2197-2.pdf. 10.1186/s13071-017-2197-2 PMC5450200 28558839

[11] Wei M, Wang L, Wu T, Xi J, Han Y, Yang X, Zhang D, Fang Q, Tang B (2016). NLRP3 activation was regulated by DNA methylation modification during mycobacterium tuberculosis infection. Biomed Res Int.

[12] Tong ML, Zhang HL, Zhu XZ, Fan JY, Gao K, Lin LR, Liu LL, Li SL, Lin HL, Lin ZF (2017). Re-evaluating the sensitivity of the rabbit infectivity test for Treponema pallidum in modern era. Clin Chim Acta.

[13] Zhang S, Liang X, Zheng X, Huang H, Chen X, Wu K, Wang B, Ma S (2014). Glo1 genetic amplification as a potential therapeutic target in hepatocellular carcinoma. International Journal of Clinical & Experimental Pathology.

[14] Frunza-Stefan S, Acharya G, Kazlouskaya V, Vukasinov P, Chiou Y, Thet Z. Immune reconstitution inflammatory syndrome associated with secondary syphilis. Int J STD AIDS 2017;28(3):302-305. http://journals.sagepub.com/doi/abs/10.1177/0956462416664469?url_ver=Z39.88-2003&rfr_id=ori:rid:crossref.org&rfr_dat=cr_pub%3dpubmed. 10.1177/0956462416664469 27566775

[15] Sell S, Gamboa D, Bakerzander SA, Lukehart SA, Miller JN (1980). Host response to Treponema pallidum in intradermally-infected rabbits: evidence for persistence of infection at local and distant sites. J Invest Dermatol.

[16] Steinbach EC, Plevy SE (2014). The role of macrophages and dendritic cells in the initiation of inflammation in IBD. Inflamm Bowel Dis.

[17] Seider K, Gerwien F, Kasper L, Allert S, Brunke S, Jablonowski N, Schwarzmuller T, Barz D, Rupp S, Kuchler K (2014). Immune evasion, stress resistance, and efficient nutrient acquisition are crucial for intracellular survival of Candida Glabrata within macrophages. Eukaryot Cell.

[18] Kaku Y, Imaoka H, Morimatsu Y, Komohara Y, Ohnishi K, Oda H, Takenaka S, Matsuoka M, Kawayama T, Takeya M (2014). Overexpression of CD163, CD204 and CD206 on alveolar macrophages in the lungs of patients with severe chronic obstructive pulmonary disease. PLoS One.

[19] Wree A, Eguchi A, McGeough MD, Pena CA, Johnson CD, Canbay A, Hoffman HM, Feldstein AE (2014). NLRP3 inflammasome activation results in hepatocyte pyroptosis, liver inflammation, and fibrosis in mice. Hepatology.

[20] Mantovani A (2013). The faces of macrophage activation. Eur J Clin Investig.

[21] Peeling RW, Hook EW (2006). The pathogenesis of syphilis: the great mimicker, revisited. J Pathol.

[22] Schroder K, Zhou RB, Tschopp J (2010). The NLRP3 Inflammasome: a sensor for metabolic danger?. Science.

[23] Zhao S, Zhu W, Xue S, Han D (2014). Testicular defense systems: immune privilege and innate immunity. Cell Mol Immunol.

[24] Marra C, Sahi S, Tantalo L, Godornes C, Reid T, Behets F, Rompalo A, Klausner JD, Yin Y, Mulcahy F (2010). Enhanced molecular typing of treponema pallidum: geographical distribution of strain types and association with neurosyphilis. J Infect Dis.

